# Psychiatric Comorbidities in 1p36 Deletion Syndrome and Their Treatment—A Case Report

**DOI:** 10.3390/ijerph182212064

**Published:** 2021-11-17

**Authors:** Wolfgang Briegel

**Affiliations:** 1 Department of Child and Adolescent Psychiatry, Psychosomatics and Psychotherapy, Leopoldina Hospital, 97422 Schweinfurt, Germany; wbriegel@leopoldina.de; Tel.: +49-9721-720-3370; 2 Department of Child and Adolescent Psychiatry, Psychosomatics and Psychotherapy, University of Würzburg, 97080 Würzburg, Germany

**Keywords:** 1p36 deletion syndrome, oppositional-defiant disorder, attention deficit/hyperactivity disorder, parent-child interaction therapy (PCIT), melatonin, methylphenidate, off label use, case report

## Abstract

1p36 deletion syndrome represents the most common terminal deletion observed in humans. Major clinical findings comprise developmental delay/intellectual disability, poor or absent expressive language, congenital central muscular hypotonia, brain anomalies, brachydactyly/camptodactyly, short feet, and characteristic facial features like straight eyebrows, deep-set eyes, and midface hypoplasia. So far, there is very limited knowledge about comorbid psychiatric disorders and their effective treatment in this special population. To fill this gap, this case report presents an initially four-year-old girl with 1p36.33–1p36.32 deletion, moderate intellectual disability, insomnia, oppositional-defiant disorder and attention deficit/hyperactivity disorder covering a period of time of about 1.5 years comprising initial psychological/psychiatric assessment, subsequent day clinic/outpatient treatment (amongst others including off-label use of melatonin and methylphenidate as well as parent-child interaction therapy) and follow-up assessment. Follow-up results indicated good efficacy of melatonin and methylphenidate medication without any adverse effects. Multidisciplinarity in diagnosis and treatment are mandatory to meet needs of patients with complex genetic disorders like 1p36 deletion syndrome. Off-label use of melatonin (for insomnia) and methylphenidate (for attention deficit/hyperactivity disorder) should be considered in young children with 1p36 deletion syndrome if behavioral interventions are not sufficient.

## 1. Introduction

Deletions of chromosome 1p36 show an incidence of approximately 1 in 5000 newborns, making 1p36 deletion syndrome (1p36 DS) one of the most commonly observed deletion disorders and the most frequent terminal deletion in humans [1,2,3,4,5]. The reasons for both this relatively high population frequency and the fact that females seem to be affected significantly more often than males are unclear [2]. In contrast, it is well documented that the majority of cases (about 52%) are pure terminal deletions that occur *de novo* [5]. Further classes of rearrangements are: interstitial deletions (29%), complex chromosomal rearrangements (12%), e.g., 1p36 deletion with a 1p36 duplication, and a derivative chromosome 1 with the 1p telomeric region replaced by another chromosome portion (7%) [5] (p. 254). Interestingly, 1p36 deletions mostly occur on the maternal chromosomes [2,5]. However, no obvious differences in phenotypic expression could be found based on parent-of-origin studies [2]. Deletion sizes of subjects with monosomy 1p36 were found to be quite variable, thus contradicting a common deletion mechanism and explaining variable phenotypic features [2]. Other possible reasons for phenotypic variability are deletion location (e.g., terminal versus interstitial), incomplete penetrance, as well as epigenetic and stochastic factors [2,4]. Several genes have been identified in the most commonly deleted 1p36.33–1p36.32 band, which is the most gene-rich part of 1p36 [5]. 

Typical clinical features of subjects with 1p36 DS (OMIM: #607872) include developmental delay (DD)/intellectual disability (ID), poor or absent expressive language, congenital central muscular hypotonia, brain anomalies (among them polymicrogyria, enlargement of the fronto-temporal opercula, cortical or generalized atrophy and prominent ventricles), seizures, eye/vision problems, sensorineural hearing loss, orofacial clefting, congenital heart defects, cardiomyopathy, renal anomalies, brachydactyly/camptodactyly, short feet, and characteristic facial features [2,3,4,5]. The facial phenotype comprises straight eyebrows, deep-set eyes, broad nasal root/bridge, midface hypoplasia, long philtrum, and pointed chin [2,3,4,5]. However, no consensus diagnostic criteria have been established until now [5].

Several critical regions for typical clinical features have been postulated so far, among them (chr.1:834,101–1,770,699) for seizures [6]; (chr1:1–2,186,829) for cleft palate [6]; (chr1:1–2,418,935) for congenital heart defects [7]; (chr1:1,916,569–3,429,762) for sensorineural hearing loss [8]; (chr1:1,916,589–3,429,762) for cardiomyopathy [8]; (chr1:2,424,876–2,425,918) for large, late-closing anterior fontanels [2]; and (chr1:4,631,608–4,643,481) for hypothyroidism [3]. Moreover, there is an increasing number of candidate genes that have been proposed to contribute to distinct phenotypes comprising MMP23B, MASP2, GABRS, SKI, PRDMI6, KCNAB2, RERE, UBE4B, CASZI, CHD5, PDPN, SPEN, ECEI, HSPG2, and LUZPI (for an overview see [4,5]).

Although the primary focus of studies and case reports on 1p36 DS has been on genetic and physical aspects so far, developmental and intellectual aspects have also been covered by some studies. In fact, DD/ID consistently seem to be the most frequent finding (almost 100% of all affected individuals) and one of the most challenging issues in subjects with the deletion [3,9]. Battaglia and colleagues reported that all 60 1p36 DS patients of their study exhibited global DD, and 52 subjects whose cognitive profile was assessed with psychological tests showed ID (about 88% severe to profound, and about 6% moderate or mild, respectively) [3].

Additionally, they found expressive language to be absent in about 75% of patients, limited to a few isolated words in 17%, and at the level of two word phrases in 8% [3]. Similarly, poor expressive language has been reported by other studies [9,10]. Overall, speech/language development seems to be significantly more impaired than motor development [9]. With regard to motor development, both fine and gross motor deficits have been found [3,5,10]. While the largest study to date found that only 26% of patients were able to walk independently (range 2 to 7 years) [3], Shim et al. reported that 46.7% were not able to walk after the age of 4 years [10]. Fortunately, both motor and language skills seem to improve over time [3,11]. Through an anonymous electronic survey, Brazil et al. were able to recruit 40 patients with 1p36 DS between 12 and 46 years old of whom 44% were reported to use complex speech abilities. Moreover, 80% were reportedly walking for at least short distances [11]. With regard to toilet training, less than 50% of subjects with the deletion seem to be toilet trained to various degrees [5].

Although global DD/ID are well-known risk factors for the development of psychiatric disorders [12,13], no specific comorbid psychiatric disorders based on criteria of the Diagnostic and Statistical Manual (DSM, e.g., [14]) and standardized psychiatric assessment have been described until now. One reason for this might be that it can be difficult to specify behavioral problems in patients with severe to profound ID [10]. Nevertheless, some studies have reported about significant stereotyped and self-abusive/self-injurious behavior among subjects with 1p36 DS [3,9,10]. Specifically, Shapira et al. reported about abusive behaviors (including hand biting, banging or throwing objects, striking people, and violent physical activity) in 5 out of 9 patients with pure 1p36 deletion [9]. Battaglia et al. found “behavior disorders” to be present in 28 out of 60 patients with 1p36 DS [3]. In detail, they reported about self-biting (30%), temper tantrums (22%), reduced social interaction (52%), stereotypies (34%), tendency to smell or beating or rolling objects in a repetitive and purposeless way (10%), and hyperphagia (13%). In a recent study including 15 patients ages 4.1 to 21.1 years at the last follow-up, two patients were found to show “Rett syndrome-like behavioral features”, i.e., severe ID with hand automatism, bruxism, and abnormal breathing pattern [10] (p. 3). Both patients did not show any regression and had 1p36 deletions of more than 9.7 Mb. Two additional patients were reported to show aggressive behavior or “attention deficit hyperactivity disorder” (ADHD) [10] (p. 3). However, no information was given regarding assessment instruments used for ADHD diagnosis. Given the fact that approximately 40% of subjects with severe ID show challenging behaviors such as self-injury and aggression and that the association between repetitive behaviors, self-injury and aggression is common across different causes of ID [15], the frequency of behavior issues in 1p36 DS described so far is in the expected range. However, these behavior issues have not been assigned to a specific psychiatric diagnosis in subjects with 1p36 DS until now, e.g., autism spectrum disorder (ASD). Interestingly, genome-wide linkage analysis in multiply-affected pedigrees with childhood-onset obsessive-compulsive disorder (OCD) comprising 245 subjects identified region 1p36.33–1p36.32 as the strongest linkage finding for OCD (heterogeneity logarithm of odds = 3.77) [16]. Stereotypies and compulsive behaviors share phenomenological similarities, however OCD symptoms have not been reported in 1p36 DS so far. Last, but not least, a significant common linkage locus was found for ADHD at chromosome 1p36 with a locus-specific heritability of 5.1% and a genome-wide empirical *p* < 0.04 [17].

To sum it up, there is a serious lack of information about psychiatric disorders in patients with 1p36 deletion and their effective treatment. This is the first report on psychiatric comorbidities in a patient with a terminal 1p36 deletion and their treatment describing initial psychological/psychiatric assessment, subsequent treatment, and follow-up assessment. The whole case report covers a period of time of about 1.5 years.

## 2. Case Presentation

Initial assessment followed the actual German child and adolescent psychiatry and psychotherapy guidelines for preschool children [18]. However, IQ testing was impossible due to a profound lack of cooperation.

### 2.1. Patient Presentation

The patient was a 4 years and 4 months old Caucasian girl of high socioeconomic status and a rural upbringing who was referred for psychiatric evaluation to the child psychiatry outpatient clinic. Her parents reported significant sleep problems (falling asleep only in the presence of her parents, restless sleep, very frequent nocturnal awakenings (up to nine times a night) with a long latency before falling asleep again, intensive dreams and nightmares, frequent screaming during the night, fatigue in the morning). Additionally, they described a very oppositional and stubborn behavior towards the parents and her older sister with frequent temper tantrums including aggressive behavior (both verbally and/or physically) or whining when she did not get what she wanted. High levels of parenting stress were reported by both parents, but especially by the girl’s primary caregiver, her mother, due to highly time-consuming care and support in routine activities as well as the girl’s challenging behaviors at home. Similarly, intensive support was needed at the girl’s childcare center which she attended five hours daily.

### 2.2. Personal History and Family Environment

The patient was born at 38 + 3 weeks of gestation via C-section to a 27-year-old, gravida 2, para 2 mother who had been prescribed venlafaxine (37.5 mg per day) and fluvoxamine (25 mg per day) during pregnancy due to depression. Birth weight (2510 g, 4th percentile), height (48 cm, 8th percentile) and head circumference (33 cm, 11th percentile) were low. Apgar scores were 9 at 1 min and 10 at 5 min. Breastfeeding had not been possible due to the mother’s medication, and the intake of solid food had always been difficult for the girl, who still had problems swallowing.

Bilateral sensorineural hearing loss with moderate hearing loss was diagnosed at the age of 5 months. Consecutively, the patient was treated with hearing aids. Subsequent examinations like electroencephalography, electrocardiography, echocardiography, and abdominal ultrasound did not reveal any further abnormalities. Global DD was first diagnosed at the age of 11 months with the German adaptation of the Bayley Scales of Infant Development (BSID-II) [19], which revealed the developmental status of a 6 months old. Cranial MRI examination under general anesthesia or sedation had been refused by the patient’s parents due to concerns regarding adverse effects of general anesthesia and sedation. A screening for inherited metabolic disorders did not give any hint for possible causes of global DD. While genetic examination at the age of 19 months revealed dysmorphic features (e.g., midface hypoplasia) without clear assignment to a genetic syndrome, karyotype and microarray analyses (CGX-HD Array, Perkin Elmer) showed the following abnormal karyotype: arr(hg19) 1p36.33p36.32(1,841,816-5,007,787)x1. This represents a terminal deletion of 3.17 Mb at the short arm of chromosome 1 comprising more than 41 genes. As both parents refused to participate in molecular analyses because they could not see their benefit, it remained unclear whether the patient’s chromosomal aberration was due to parental rearrangements or a de novo aberration.

The patient was able to walk independently by the age of 17 months, but she could not speak first words before the age of 24 months. At the age of 1.5 years, she had febrile spasms, but she never developed seizures. The girl attended daycare since the age of 18 months without any separation problems, but she soon needed additional individual care (20 h a week). With that support she could be integrated quite well although—according to her teacher—she had significant language and motor deficits, a very short attention span and a low frustration tolerance. The patient had received curative education since the age of 15 months, whereas speech and occupational therapy followed later on. She was still wearing a diaper as she showed daytime wetting, soiling and bedwetting. At clinical presentation in the outpatient clinic, the parents reported no stressful events in their child’s life, and the girl did not receive any medication.

The patient’s parents lived together and were not consanguineous. Her mother had graduated from German ”Gymnasium“ (grades 5–13) as academic secondary school, and was a trained industrial clerk and housewife. The girl’s 37 year-old father had graduated from university with a MD and was working fulltime at a hospital. While he reported no history of physical or mental disorders, the patient’s mother described severe problems with her family of origin and depressive episodes. The patient had an elder sister who was 6 years old and went to elementary school. According to her parents the patient’s sister had no history of physical or mental problems.

### 2.3. Pretreatment Assessment

#### 2.3.1. Child Aspects

##### Direct Observations

On physical examination, the patient was cooperative, showed normal eye-contact and social smiling and was in good general condition, with normal height (55th percentile) and weight (24th percentile) but microcephaly (47 cm, below 3rd percentile). The following dysmorphic features could be found: midface hypoplasia with broad nasal root/bridge, straight eyebrows, and brachydactyly. Additionally, she showed muscular hypotonia and was wearing two hearing aids due to a hearing loss.

By means of unstructured psychiatric interviews with the patient’s parents and her teacher severe developmental delays, insomnia, oppositional-defiant behavior problems and attention-deficit/hyperactivity symptoms both at home and at the daycare center became apparent. During mental examination (based on the Clinical Assessment Scale for Child and Adolescent Psychopathology-D [20]), the patient appeared younger than her real age. She was in a good mood, friendly and cooperative, but overly affectionate. Although she was able to speak 3-word phrases, significant language deficits and echolalia became apparent. Additionally, the patient showed a very short attention span, significantly increased impulsivity and psychomotor hyperactivity. Neither motor stereotypies nor self-abusive or OCD behaviors could be observed.

The German adaptation of the Movement Assessment Battery for Children-2 (M-ABC-2 [21]), a validated measure to assess motor skills in children ages 3 to 16 years indicated significant deficits in all domains (results are given as percentiles): dexterity: 1; ball skills: 2; balance: 1; total: 0.4. The Test zur Überprüfung des Grammatikverständnisses (TROG-D [22]), a validated German measure to assess receptive grammar skills in children ages 3 to 10 years, revealed a *t*-score of 29 indicating severe deficits. The Heidelberger Marschak Interaction Method [23], a semi-structured instrument to analyze dyadic parent-child interactions showed significant delays in cognitive, verbal and motor development, significant oppositional child behaviors, especially towards the mother, a very low frustration tolerance and a very short attention span.

##### Questionnaires

The Child Behavior Checklist/1.5-5 (CBCL/1.5-5 [24]), a widely used parent-report questionnaire which consists of 100 items constituting seven syndrome scales (emotionally reactive, anxious/depressed, somatic complaints, withdrawn, sleep problems, attention problems, aggressive behavior) and three global scales (internalizing problems, externalizing problems, total problems) was used to assess a broad spectrum of behavior problems. Each item of the CBCL/1.5-5 can be scored as 0 = ‘not true as far as you know’, 1 = ‘somewhat or sometimes true’, or 2 = ‘very true or often true’. Results are given as *t*-scores (mean *t*-score: 50, standard deviation: 10). *T*-scores ≥ 70 for syndrome scales as well as *t*-scores ≥ 64 for global scales are classified as in the “clinical range”. *T*-scores from 65 to 69 for syndrome scales and from 60 to 63 for global scales represent the so-called borderline clinical range. A German validity study could largely replicate the original factor structure [25], but the German questionnaire still awaits a standardization study with a representative sample. Therefore, American norms had to be used. The CBCL/1.5-5 [24] revealed clinical scores for the following scales: sleep problems (*t =* 88), attention problems (*t* = 70), aggressive behavior (t = 79), externalizing problems (*t* = 77) and total problems (*t* = 74). The scale emotionally reactive reached a score in the borderline clinical range (*t* = 65). Clinically significant disruptive behavior problems (Intensity Scale: 207, *t*-score: > 96; Problem Scale: 28, *t*-score: 84) were indicated by the German version of the Eyberg-Child Behavior Inventory (ECBI; [26]), a well-validated parent-report questionnaire comprising 36 items to assess disruptive behaviors of 2- to 9-year-old children on the intensity scale (representing the parents’ perception of behavior frequency on a range from (1) never to (7) always) and the problem scale (indicating whether a behavior is considered problematic or not; YES/NO answer). Similarly, the Fremdbeurteilungsbogen für den Störungsbereich Störungen des Sozialverhaltens (FBB-SSV [27]), a validated German measure consisting of 23 items describing symptom criteria of oppositional-defiant disorder (ODD), conduct disorder (CD) and disruptive mood dysregulation disorder (both ICD-10 and DSM-V criteria), showed results conspicuous for ODD. Items can be rated by parents or teachers of children ages 4 to 17 years on a 4-point answer scale ranging from not at all (0) to particularly (3). Parent results are given as stanine scores (mean stanine score: 5, standard deviation: 2) whereas teacher ratings can be classified as normal, slightly abnormal, abnormal and highly abnormal. The FBB-SSV was filled out by the patient’s parents (stanine values; oppositional-defiant behavior: 9; aggressive-dissocial behavior: 9; total score: 9) and her teacher (oppositional-defiant behavior: abnormal; aggressive-dissocial behavior: slightly abnormal; total score: slightly abnormal). The German ADHD rating scale Fremdbeurteilungsbogen ADHS im Vorschulalter (FBB-ADHS-V [27]) which comprises 20 items describing the symptom criteria according to both the ICD-10 and DSM-V, was given to the patient’s parents and her teacher. Items of this questionnaire can be rated by parents or teachers of children ages 3 to 6 years on a 4-point answer scale ranging from not at all (0) to particularly (3), with higher scores indicating greater ADHD-related behavior. Mean item scores can be calculated for the two DSM-V dimensions inattention and hyperactivity-impulsivity. It is also possible to classify respondents with regards to the criteria for ADHD subtypes as defined by the DSM-V. Results are given as stanine scores (mean stanine score: 5, standard deviation: 2). Both attention deficits and hyperactivity-impulsivity (stanine values; parent ratings: attention deficit: 9; hyperactivity-impulsivity: 8; total ADHD score: 9; teacher ratings: attention deficit: 9; hyperactivity-impulsivity: 9; total ADHD score: 9) scored high on the the FBB-ADHS. Last, but not least, elevated scores for total behaviour problems (98th percentile, total ID sample), disruptive/antisocial problems (98th percentile); communication disturbance (98th percentile) and anxiety problems (86th percentile) were indicated by the German version of the Developmental Behaviour Checklist (VFE [28], parent report form. This broad-spectrum questionnaire comprises 96 items to assess behavioral and emotional problems of 4–18-year-old subjects with developmental and intellectual disabilities. Each item can be scored as 0 = ‘not true as far as you know’, 1 = ‘somewhat or sometimes true’, or 2 = ‘very true or often true’. Results can be given as *t*-scores or percentiles for four different groups of subjects with ID: mild, moderate, severe or the total ID sample.

#### 2.3.2. Parent Aspects

The Social Orientation of Parents with Handicapped Children (SOEBEK) [29], a validated questionnaire to assess stress and coping strategies among caregivers of 1–14-year-old German subjects with mental and/or physical disability, was filled out by both parents. The SOEBEK comprises the following scales: partnership intensification (6 items), ability to meet own needs (5 items), use of social support (6 items), focusing on the child with a disability (6 items), and parental stress (20 items). Items of the coping strategies are rated on a 6-point Likert scale from never (1) to very often (6), whereas most items of parental stress have a 5-point answer scale ranging from 1 (very seldom) to 5 (very often). Results can be given as percentiles for either children with physical handicaps or children with physical and mental handicaps. Parental stress and focusing on the child with a disability scores above the 84th percentile are considered to be clinical whereas scores of partnership intensification, ability to meet own needs, and use of social support are classified as in the clinical range when they are below the 16th percentile. The SOEBEK revealed high levels of parental stress (mother: 90th percentile; father: >95th percentile). Only the patient’s father showed significantly increased focusing on the child (95th percentile; mother: 70th percentile), but both parents reported about a reduced ability to meet own needs (father: 5th percentile; mother 5th to 10th percentile). To screen for parental psychopathology, both parents filled out the Mini-Symptom-Checklist (Mini-SCL [30]), a well validated German self-report measure comprising 18 items to assess depression (6 items), anxiety (6 items) and somatization (6 items) during the last seven days in subjects from the age of 12 years. Items can be rated on a 5-point answer scale ranging from not at all (0) to extreme (4). Scores of all items can be summed to obtain the global severity index. Results are given as *t*-scores. The patient’s mother showed elevated scores (*t* ≥ 60) for all scales (depression: *t* = 61; anxiety: *t* = 62; somatization: *t* = 66; total: *t* = 64). Similarly, the father scored high on the following scales: anxiety: *t* = 66; total: *t* = 62. The Satisfaction with Life Scale (SWLS [31]) indicated a significantly reduced maternal satisfaction with life (*t* = 34) while the paternal score was in the normal range (*t* = 47).

### 2.4. Diagnoses and Recommendations

Assessment results were consistent with the DSM-V diagnosis of global developmental delay with significant delays in fine and gross motor skills, expressive and receptive language, cognition and socio-emotional development (DSM-V: 315.8; main diagnosis). Additionally, the patient fulfilled diagnostic criteria for insomnia (DSM-V: 307.42) and showed clinically significant symptoms of ODD (DSM-V: 313.81) and ADHD-combined type (DSM-V: 314.00). Compared with the girl’s developmental age, ADHD symptoms were clearly abnormal. Similarly, ODD symptoms were significantly in the clinical range compared with the ID population.

As there are no specific guidelines regarding the management and treatment of psychiatric disorders in patients with 1p36 DS recommendations followed the guidelines of the German societies of child and adolescent psychiatrists for preschool children [18]. Specifically, it was recommended that the patient attended a psychiatric day clinic for intensive multimodal treatment comprising speech therapy, occupational therapy and behavior therapy for the girl, and parent management training and social counselling for the parents. If necessary, medication (e.g., melatonin) should be taken into consideration. Both parents agreed to these recommendations. Additionally, it was recommended that the mother as the primary caregiver of the patient should think about starting individual psychotherapy as she scored high for psychopathology on the Mini-SCL and reported a significantly reduced satisfaction with life.

### 2.5. Outcome Measures

#### 2.5.1. Child Measures

The German version of the Eyberg Child Behaviour Inventory (ECBI [26]) represented the main child outcome measure for disruptive behaviors. The ECBI was administered to the parents at pretreatment assessment, during PCIT treatment, at the end of the day clinic treatment and at follow-up.

The Child Behavior Checklist/1.5-5 (CBCL/1.5-5 [24]) was used as the main assessment instrument regarding sleep problems. It was administered to the patient’s parents at pretreatment assessment and at follow-up.

The German version of the Developmental Behaviour Checklist, the Verhaltensfragebogen bei Entwicklungsstörungen (VFE [28]) was used to compare behavioral and emotional problems of the patient with those in the population of 4–18-year-old subjects with developmental and intellectual disabilities. It was given to the parents at pretreatment assessment and at follow-up.

The Fremdbeurteilungsbogen für den Störungsbereich Störungen des Sozialverhaltens (FBB-SSV [27]) was administered to the patient’s parents and her teacher at pretreatment assessment and at follow-up to have different sources of information.

Similarly, the Fremdbeurteilungsbogen ADHS im Vorschulalter (FBB-ADHS-V [27]) was filled out by the patient’s parents and her teacher at pretreatment and at follow-up assessment allowing to compare the perception of different informants from different settings.

#### 2.5.2. Parent Measures

The Social Orientation of Parents with Handicapped Children questionnaire (SOEBEK [29]) was filled out by both parents at pretreatment and follow-up assessment to gather information on parental stress and coping strategies compared to other caregivers of children with mental and/or physical disability. A significant positive correlation has been found between parental stress and the total score of the Parenting Stress Index [32,33].

To screen for parental psychopathology, the Mini-Symptom-Checklist (Mini-SCL [30]) was given to the patient’s parents at pretreatment assessment and at follow-up.

To determine whether treatment effects as assessed with outcome measures were meaningful or due to random error, reliable change indices (RCIs) [34] were calculated for all outcome measures which were in the clinical range at pre-treatment assessment and for which test-retest-reliability coefficients (*r*_xx_) were available. Specifically, the following coefficients were used: 20-month *r*_xx_’s for the VFE [28] (p. 37), 12-month *r*_xx_’s for the American CBCL/1.5-5 [35] (p. 80), 12-month *r*_xx_’s for the SOEBEK [29], (p. 17). There are no German test–retest reliability results for the ECBI so far, thus Dutch 6-month *r*_xx_’s [36] were used. No published test-retest-reliability coefficients were available for the FBB-ADHS-V, the FBB-SSV and the Mini-SCL.

RCI scores > 1.96 indicate that the change between pre-treatment scores and post-treatment/follow-up scores is not due to random error (*p* < 0.05). As the RCI alone does not indicate clinical significance, the following criteria were applied to assess clinical significance: (1) the pre-treatment score is in the clinically significant range; (2) the post-treatment/follow-up score is in the normal range; (3) the change in scores is statistically reliable as defined using the RCI [37].

### 2.6. Course of Treatment

Day clinic treatment which had to be interrupted for 4 weeks due to SARS-COVID-19 pandemic restrictions comprised a highly structured daily routine based on the principles of the evidence-based parent management programs Positive Parenting Program (Triple P [38]) and Parent-Child Interaction Therapy (PCIT [39]), daily interventions by educationalists to facilitate community skills, individual psychotherapy (twice a week), speech therapy (once or twice a week), and occupational therapy (individual and group, twice a week). During the first two weeks of treatment, parents had social counselling and were coached to establish good sleep hygiene. As sleep hygiene interventions were not sufficient, parents were informed about the possibility of Slenyto^®^ (prolonged-release melatonin) off-label use. Slenyto^®^ is approved in Europe, Japan and the US for the treatment of insomnia in subjects aged 2 to 18 years with autism spectrum disorders and/or Smith-Magenis syndrome where sleep hygiene measures have been insufficient. This medication has been shown to improve sleep latency, sleep maintenance and total sleep time, to improve child’s externalizing behaviors (hyperactivity/inattention and conduct), and to improve parents’/caregivers’ quality of life [40]. After both parents had given informed consent to an off-label trial and laboratory tests (including ferritin) had revealed regular results, medication was started on treatment day 14 with 1 mg daily. By increasing the dose to 2 mg a day, parents reported that their daughter’s sleep normalized completely without any adverse effects. However, oppositional behavior problems as well as ADHD behaviors did not change at all, thus causing persistently high levels of parental stress. As psychosocial interventions, especially parent management trainings, are recommended as first-line treatment for preschool children with disruptive behavior disorders (e.g., [41,42]), PCIT [39] was started. This evidence-based, manualized parent management program has been designed to help parents develop an authoritative parenting style. It combines both play therapy and behavioral therapy approaches, and has been found effective among children with DD and ID [43]. During the first phase of PCIT, the child-directed interaction (CDI), parents are coached to learn the “PRIDE” skills (P for (labeled) praise, R for reflection, I for imitation, D for behavioral description and E for enjoyment) and to avoid questions, commands or criticism in order to improve their relationship with their child [44]. The second phase, which is called parent-directed interaction (PDI), can be started after parents have achieved CDI mastery i.e., 10 behavioral descriptions; 10 reflections; 10 labeled praises; and not more than three questions, commands and criticisms in total during five minutes of coding with the DPICS [45] and a significant improvement of their relationship with their child. During PDI, parents are coached how to give specific, age-appropriate, direct commands and how to proceed with positive reinforcement for compliance or a time-out procedure following noncompliance [44]. To monitor therapy effectiveness and guide coaching both the ECBI [26] and the Dyadic Parent-Child Interaction Coding System (DPICS [45]) are regularly used. PCIT is considered successfully completed when parents reach mastery of the CDI and PDI skills, the child’s ECBI intensity score is below a *t*-score of 55, and parents express confidence in managing their child’s behaviors on their own [44]. To achieve these goals, parents are requested to spend 5 min “special time” a day to exercise/use their PRIDE skills during the full course of the treatment. Additionally, PDI homework includes giving commands and follow-through.

DPICS baseline coding showed that the girl’s parents used significantly more Don’t Skills (parental commands, questions and criticism) than Do Skills (parental praises, behavior descriptions and reflections; for further information on CDI results see Table 1). After having attended a CDI teaching session both parents continued with coaching sessions. Specifically, the patient’s mother participated in six CDI coaching sessions, two of them together with her husband who attended a total of 4 CDI sessions with coaching being conducted through a one-way mirror using a “bug in the ear” device. In addition to standard PCIT, parents were taught to increase their use of gestures and visualizations.

At the end of the day clinic treatment, the patient’s mother reached criteria to proceed to PDI (see Table 1).

The patient was finally discharged on the 52nd day clinic day representing a shorter length of stay than usual (*n* = 75 days). At this time, her parents reported that the girl’s sleep had completely normalized. They also gave the feedback that the patient’s social behavior had improved, but was still very challenging. ECBI results were still in the clinical range (*t*-scores; mother: intensity score: 81, problem score: 71; father: intensity score: 78, problem score: 63). RCIs > 1.96 could be demonstrated for ECBI Intensity scores of both parents (mother: 3.840; father: 4.458), and the paternal ECBI Problem score (mother: 1.505; father: 2.422).

As it had become increasingly clear during day clinic treatment that the patient fulfilled criteria of moderate ID, parents were informed about this diagnosis. They were advised to continue with Slenyto^®^ medication and “special time” and to start with the PDI part of PCIT during outpatient treatment. The parents agreed with these recommendations and were also willing to continue with speech therapy, occupational therapy and individualized support at the daycare center. Additionally, they were informed about possible off-label pharmacotherapy approaches to reduce disruptive behavior problems (e.g., stimulants and neuroleptics), but did not want an additional medication at that time.

As it turned out after the end of the day clinic treatment, the parents decided not to continue with PCIT because they did not have enough time. They also were not able to do “special time” with their girl on a regular basis. For similar reasons, the mother had not started psychotherapy so far. Three months after discharge the parents informed the therapist that attention problems had become an increasing problem at the daycare center. Therefore, they asked for a medication with stimulants for their now 5 years and 4 months old girl. According to the parents’ will, medication should cover the time from 08:30 a.m. to 02:30 p.m. during daycare days. As medication was clinically indicated, the patient’s parents were thoroughly informed about methylphenidate, a reuptake-inhibitor of dopamine and norepinephrine, which is widely used for ADHD since the 1990s. Methylphenidate-containing medicines are approved in many countries for the treatment of children aged six years or older and adolescents with ADHD as part of comprehensive treatment programs. After the parents had given informed consent to an off-label trial and laboratory tests as well as electrocardiogram had revealed regular results, immediate-release methylphenidate was finally started at a daily dose of 2.5 mg in the morning. By increasing the dose to 5 mg, the parents reported positive feedback from the daycare center. Medication seemed to cover the requested period of time without any adverse effects. The patient’s parents were so satisfied that they decided to continue with the methylphenidate medication without any changes. 4 weeks later, parents sent back follow-up outcome measures.

### 2.7. Follow-Up Assessment

#### 2.7.1. Child Measures

All parent-report questionnaires indicated clinically relevant disruptive behavior problems. Specifically, the following scales were in the clinical range: CBCL/1.5-5 *(t*-scores): attention problems: 73, aggressive behavior: 70, external problems: 73, total problems: 67; ECBI (*t*-scores): intensity scale: 89; problem scale: 71); FBB-SSV (stanine values): oppositional-defiant behavior: 9; aggressive-dissocial behavior: 9; total score: 9; FBB-ADHS-V (stanine values): attention deficit: 9; hyperactivity-impulsivity: 9; total ADHD score: 9; and *VFE* (percentiles): total behavior problem score: 90th percentile; disruptive/antisocial: 92th percentile. In contrast, the scores for the CBCL/1.5-5 scales sleep problems (*t*-score: 64) and emotionally reactive (*t*-score: 51) were now in the normal range.

Teacher ratings were normal for all FBB-SSV scales, whereas the FBB-ADHS-V revealed significant attention deficit problems (stanine score: 9), but normal scores for hyperactivity-impulsivity (stanine score: 5). Total ADHD stanine score was 7.

#### 2.7.2. Parent Measures

The SOEBEK still showed elevated levels of maternal but not of paternal stress (95th vs. 70th percentile). While the mother did not show increased focusing on the child (45th–50th percentile), the patient’s father was near the cut-off (80th–85th percentile). Both parents still reported about a reduced ability to meet own needs (<16th percentile; father: 1st to 3rd percentile; mother: 15th percentile).

The Mini-SCL (Franke, 2017) revealed no elevated scores (*t* > 60) for the patient’s father (depression: *t* = 51; anxiety: *t* = 50; somatization: *t* = 49; total: *t* = 49) whereas the mother had high scores for depression (*t* = 67) and overall psychological stress (*t* = 61). Scores for anxiety (*t* = 54) and somatization (*t* = 40) were in the normal range.

#### 2.7.3. Reliable Change Index and Clinically Significant Change

Regarding outcome measures at follow-up (under treatment with 2 mg melatonin and 5 mg methylphenidate [for 8 weeks]) for which test-retest reliability coefficients were available (VFE, CBCL/1.5-5, and SOEBEK), only the CBCL/1.5-5 scale sleep problems showed a RCI > 1.96 (RCI: 2.01). Applying the Jacobson, Roberts, Berns, and McGlinchey criteria [37], the reduction of sleep problems was clinically significant (pre-treatment *t*-score: 88, *t*-score under treatment: 64).

Although no RCIs could be calculated for the FBB-ADHS-V and the FBB-SSV, teacher ratings suggested clinically significant reductions in hyperactivity-impulsivity (stanine scores; pretreatment: 9, final assessment: stanine 5) as well as a normalization of ODD and CD behaviors at the daycare center. In contrast, parent ratings did not show essential changes and were still in the clinical range. Regarding psychological well-being of the patient’s parents as measured with the Mini-SCL, symptom reductions of at least 1.25 standard deviations from clinical to normal scores could be found for the father’s anxiety scale and his global symptom index thus suggesting clinically significant changes.

## 3. Discussion

This case report deals with a girl with a terminal deletion of 3.17 Mb at the short arm of chromosome 1 who was four years old at her first presentation at the child and adolescent outpatient clinic. She presented with the following abnormal physical conditions: muscular hypotonia, microcephaly, bilateral sensorineural hearing loss. Dysmorphic facial features comprised midface hypoplasia with broad nasal root/bridge, straight eyebrows, and brachydactyly. Results of psychiatric and psychometric assessment indicated global DD/moderate ID with comorbid insomnia, ODD, and ADHD-combined type. Treatment consisted of 52 days at a child psychiatry and psychotherapy day clinic and subsequent outpatient treatment for nine months. Day clinic treatment comprised a highly structured daily routine based on the principles of PCIT and PPP, interventions by educationalists, speech and occupational therapists, individual psychotherapy, and prolonged-release melatonin (Slenyto^®^) medication for the patient as well as counseling and the CDI phase of PCIT (with the patient and her parents), which altogether resulted in clinically significant reductions of sleep problems and fewer disruptive behavior problems in the family setting. The subsequent outpatient treatment included continuing melatonin medication and an off-label trial of immediate-release methylphenidate. At follow-up, the patient’s sleep was within the normal age range whereas disruptive behaviors in the family setting had become worse again, and the patient’s mother still showed elevated levels of stress and depression. In contrast, disruptive behavior problems at the daycare center showed a significant reduction suggesting a good effect of low dose methylphenidate medication. No adverse effects could be found under melatonin and methylphenidate off-label medications.

This is the first detailed report on psychiatric disorders in 1p36 DS and their treatment. Until now, behavior problems, e.g., stereotyped and self-abusive/self-injurious behaviors or temper tantrums/aggression have been reported in several studies [3,9,10]. However, they have not been assigned to DSM-based psychiatric disorders, e.g., ASD, stereotypic movement disorder (SMD) or ODD. Similarly, one patient was described as having ADHD, but no information was given regarding assessment instruments [10]. In contrast, for the patient described in this report well-validated assessment instruments were applied, psychiatric diagnoses were assigned according to the actual DSM-V criteria, and treatment outcomes were evaluated using statistical approaches where possible.

Considering the high prevalence of 1p36 DS in the general population and the very high prevalence of DD/ID among subjects with 1p36 DS [3,4,5], it is very astonishing that there are so few reports on psychiatric disorders in this special population. For example, there is much more knowledge on behavioral phenotypes/psychiatric disorders in other genetic syndromes with comparably high or even lower prevalence rates, e.g., 22q11.2 deletion syndrome, fragile X syndrome or Williams syndrome [46,47,48]. There are some possible explanations for this paucity of knowledge in 1p36 DS. First, studies on 1p36 DS have predominantly focused on genetic and somatic aspects thus far. Second, most psychiatric disorders are not seen before the age of two years. Third, there seem to be two effects facilitating an under-diagnosis of psychiatric disorders in subjects with ID: “over-shadowing” (the tendency of clinicians to overlook additional psychiatric diagnoses after ID has been diagnosed) and “masking” (clinical characteristics of a mental disorder are masked by cognitive, language, or speech deficits) [49]. Fourth, there is a paucity of both standardized classification guidelines for identifying “normal” or “usual” levels of psychiatric symptoms in subjects with ID and instruments to assess behavior aspects which are validated for the use in subjects with ID, especially children and adolescents.

However, there is clear evidence that the prevalence of psychiatric disorders among children with ID significantly exceeds that of children without ID [50,51], and that the prevalence of self-injurious and stereotypic behaviors increases with the degree of ID [52,53,54]. ADHD, ODD and sleep problems, the patient’s psychiatric diagnoses, are among the most frequently diagnosed psychiatric disorders in children with ID with prevalence rates ranging from 8–39% for ADHD, 12–23% for ODD, and 24–86% for sleep problems [13,50,51,55,56]. Interestingly, neither stereotypies nor self-injurious behaviors have been a significant problem in our patient which might be due to her only moderate ID and her reduced but albeit sufficient ability to express her needs both verbally and non-verbally.

A significantly positive association between the frequency of child behavior problems, especially externalizing ones, and parental stress has been well documented [57,58], and highest stress levels are found among parents of children with ASD and DD [58]. Moreover, parenting stress levels and child behavior problems can reinforce each other mutually (e.g., [59,60]), especially in children with mental and physical health problems [58]. In accordance with these findings pre-treatment stress levels as well as symptoms of depression, anxiety and somatization were high for both parents. At follow-up maternal stress was still elevated while the father’s stress was in the normal range. Similarly, the mother’s scores for depression and overall psychological stress were in the clinical range while the father did not show elevated scores at all. Thus, interventions might have had a positive impact predominantly on paternal stress and psychological well-being.

So far, reports on the treatment of psychiatric disorders in 1p36 DS have been lacking completely. The results of this case report have some important clinical implications for the 1p36 DS population. First, prolonged-release melatonin might be very beneficial to reduce sleep disorder problems although it is not approved for this special population so far. Sleep latency, sleep maintenance and total sleep time could be improved in a clinically significant way without any adverse effects in the patient presented here. However, Slenyto^®^ medication did not have a significant effect on the girl’s externalizing behaviors (hyperactivity/inattention and conduct) or the parents’ stress level which is in contrast to findings in children and adolescents ages 2–18 years with ASD and/or Smith-Magenis syndrome [40] for which Slenyto^®^ is approved in Europe, Japan and the US. Second, methylphenidate could be helpful to reduce disruptive behavior problems in patient with 1p36 DS even when used off-label (i.e., before the age of six years). In this case report, teacher ratings suggested clinically significant reductions in hyperactivity-impulsivity as well as a normalization of ODD and CD behaviors at the daycare center under medication with a single dose of 5 mg immediate-release methylphenidate in the morning. No adverse effects have been described under this medication. These findings are in accordance with results of meta-analyses conducted in children with ADHD and normal intellectual abilities [61,62] as well as with the findings of a recent meta-analysis in children with ADHD and ID or borderline intellectual functioning [63]. This latter meta-analysis revealed significant beneficial methylphenidate effects on overall ADHD severity, conduct, hyperactivity, and inattentive symptoms, and a good methylphenidate tolerance [63]. Methylphenidate is approved as part of comprehensive treatment programs. In the case reported here, psychiatric day clinic treatment as such a comprehensive treatment program preceded methylphenidate off-label use. Third, a comprehensive treatment program seems to be indicated in most cases of young children with 1p36 DS given the very high frequency of ID and a thus elevated risk for psychiatric comorbidities. To meet all needs, such a treatment program should ideally comprise speech therapy, occupational therapy and parent management training. In the case presented here, day clinic treatment resulted in statistically significant reductions of ECBI Intensity scores of both parents as well as clear improvements in parental CDI skills. Contrary to recommendations at day clinic discharge, the parents had not continued “special time” at home nor had they started with the PDI part of PCIT which might be a reason why ECBI scores at follow-up indicated a relapse of disruptive behavior problems. As these behaviors show considerable stability without adequate interventions thus causing profound disability and family dysfunction [64,65,66], further interventions (e.g., PDI phase of PCIT, long-acting methylphenidate, additional neuroleptic medication, individual psychotherapy or psychopharmacotherapy for the patient’s mother) might be necessary in the future for the patient presented here.

This case report has clear strengths: Well standardized and carefully chosen clinically relevant measures have been used to assess child behavior problems (both with parent and teacher reports), parent-child interaction quality (by means of standardized therapist observations) and parental psychopathology and stress (by means of self-reports). However, there are also some limitations including the lack of a control group and limited generalizability due to the case study design, the lack of a standardized diagnostic interview for psychiatric disorders in children (as there is no validated German interview on psychiatric disorders in preschool children thus far), and the fact that neither standardized intelligence tests nor a cranial MRI could be conducted as the patient did not show sufficient cooperation.

## 4. Conclusions

So far, there is very limited knowledge about psychiatric disorders in patients with 1p36 DS, although this genetic condition represents the most frequent terminal deletion in humans. Moreover, reports on the effective treatment of psychiatric disorders have been lacking completely until now. Thus, this first detailed report on psychiatric comorbidities in 1p36 DS and their treatment enhances our knowledge about both aspects significantly. Specifically, multi-disciplinarity in diagnosis and treatment is mandatory to cover all somatic and psychological aspects associated with the deletion. With regard to the psychological well-being of affected subjects, regular screenings for psychiatric disorders are indicated which should be followed by thorough psychological and psychiatric assessments if screening results suggest psychiatric disorders. Interventions for psychiatric disorders should be chosen according to the general guidelines of the psychological and psychiatric scientific societies as long as there are no recommendations which are specific for 1p36 DS subjects. As this case report proves, off-label use of melatonin (for insomnia) and methylphenidate (for ADHD) should be taken into consideration if behavioral interventions are not sufficiently effective in young children with 1p36 DS. Moreover, PCIT might be a good choice to effectively reduce disruptive behaviors in this population if it can be successfully completed. Last but not least, early access to developmental therapies like speech and occupational therapy should be provided to reduce developmental delays. Further studies are needed to delineate behavioral phenotype/psychiatric disorders in patients with 1p36 DS and to evaluate therapeutic interventions.

## Figures and Tables

**Table 1:** Parents’ child-directed interaction (CDI) skills at baseline and at the end of the day clinic treatment.

DPICS *	Mother		Father	
	Baseline	End of Day Clinic Treatment	Baseline	End of Day Clinic Treatment
**Do Skills**				
Behavioral Description	0	15	0	4
Reflection	4	10	7	7
Labeled Praise	0	11	0	7
Unlabeled Praise	6	4	2	1
Total	10	40	9	19
**Don’t Skills**				
Question	23	0	22	0
Command	10	0	9	0
Criticism	2	2	1	0
Total	34	2	32	0

* There are no German reference values for DPICS codes so far.

## Data Availability

Data is contained within the article.
